# Grand Challenges in Terrestrial Microbiology: Moving on From a Decade of Progress in Microbial Biogeochemistry

**DOI:** 10.3389/fmicb.2020.00981

**Published:** 2020-05-15

**Authors:** Sara Hallin, Paul L. E. Bodelier

**Affiliations:** ^1^Department of Forest Mycology and Plant Pathology, Swedish University of Agricultural Sciences, Uppsala, Sweden; ^2^Department of Microbial Ecology, Netherlands Institute of Ecology (NIOO-KNAW), Wageningen, Netherlands

**Keywords:** carbon cycling and storage, nitrogen cycling bacteria, methane cycling, nitrification–denitrification, climate change, environmental pollution, soil microbiomes

Climate change and environmental pollution are major challenges for humanity that affect terrestrial ecosystems and multiple ecosystem services, possibly leading to irreversible changes (IPCC, 2018) and beyond tipping points (Steffen et al., 2015, 2018). There are both positive and negative feedbacks from terrestrial systems to climate and environmental change, and here microbes play a central role (Fierer, 2017). Despite the fundamental importance of microbes in the functioning of soils and our planet, they are rarely considered in global earth system models, IPCC predictions, biodiversity conservation, or even within the global sustainable development goals. Recently, a number of scientists compiled a consensus statement framework of the Alliance of World Scientists Movement (https://www.scientistswarning.org/) arguing for the recognition of the importance of microbes for the biosphere and humanity and the inclusion of microbiology in all the great societal challenges ahead (Cavicchioli et al., 2019), including the global degradation of soils and its associated consequences for food production. Soil microbial communities are central when it comes to climate and environmental change because of their fundamental role in biogeochemical cycles, especially in a large number of reactions and transformation pathways in the carbon and nitrogen cycles (Fierer, 2017). These cycles are interlinked and affect primary production, for example by determining availability of plant nutrients and by regulating the amount of net storage of organic carbon in soil and emissions of greenhouse gases like methane and nitrous oxide. The crucial role of microbial biogeochemistry in tackling the societal challenges ahead is a strong incentive to make progress. Some of the advances in microbial aspects of biogeochemical cycling in the past decade are highlighted below using examples from carbon and nitrogen cycling.

Several new pathways and major players involved in carbon and nitrogen cycling have unfolded in the past decade, underlining the challenge of linking composition and functioning of environmental microbial communities given the lack of knowledge of essential components. However, tremendous progress has been made in key “blindspots” in our field. Using traditional as well as novel metagenomics-assisted techniques in combination with a lot of patience has enabled the isolation and enrichment of novel microbes playing eminent roles in biogeochemical cycling. In the carbon cycle, the enigmatic rice cluster 1, harboring methanogens responsible for most of the methane emitted from rice paddies globally, was isolated and taxonomically described (Sakai et al., 2010), as was a methanogen (*candidatus* Methanoflorens stordalenmirensis) which was largely responsible for methane production in thawing permafrost soils (Mondav et al., 2014). In recent years, the dogma that only archaea can produce methane and only in anoxic environments was challenged, as exemplified by the recent discovery of cyanobacteria releasing methane, while producing photosynthetic oxygen (BiŽić et al., 2020) as well as methane generation from methylphosphonates by marine bacteria (Carini et al., 2014). By contrast, the degradation of methane, which was previously assumed to be largely occurring under oxic conditions in terrestrial systems, is now known to be possible without molecular oxygen by utilizing oxygen atoms generated by NO dismutation by *candidatus* Methylomirabilis oxyfera (Ettwig et al., 2010), or nitrate (Haroon et al., 2013) or iron (Ettwig et al., 2016) as electron acceptor by *candidatus Methanoperedens nitroreducens* suggesting yet unknown linkages between elemental cycles of high environmental relevance (Segarra et al., 2015). Microbes oxidizing atmospheric methane in upland soils (so called “high affinity” methanotrophs) have also been subjected to laboratory isolation and could be observed under the microscope after a hunt of almost 30 years. They turned out to be species closely related to members of the genus *Methylocapsa* being able to grow both on atmospheric levels and high concentrations of methane (Pratscher et al., 2018; Tveit et al., 2019). The organisms seem to gain energy by consuming hydrogen and CO, which then generates the reducing equivalents for atmospheric methane oxidation. This finding exemplifies radical changes of our ideas in this field and provides microbes enabling experimental elucidation of the environmental controls of this climate relevant reaction.

Fundamental leaps in understanding the role of microbes regarding CO_2_ fluxes to the atmosphere as well as soil carbon sequestration have also been achieved (Allison et al., 2010; Abramoff et al., 2018). Forest soils have been given special attention and recent studies show that mycorrhizal mycelium contributes to carbon storage by both allocating carbon into the soil and by the slow decomposition of ericoid mycorrhizal cell walls (Clemmensen et al., 2015; Fernandez and Kennedy, 2018). However, certain ectomycorrhizal fungi have been shown to actively decompose soil organic matter (Lindahl and Tunlid, 2015) and thereby contribute to carbon losses. Thus, the composition of fungi and differences in their functions in soil may have an impact on CO_2_ fluxes and carbon sequestration. In agricultural soil, the role of microbes for carbon storage and carbon stabilization is increasingly recognized (Liang et al., 2017) as is the role of fungi in this matter (Verbruggen et al., 2016). Interestingly, it seems like a greater abundance and diversity of decomposers initially leading to increased decomposition rates will result in increased soil organic carbon stocks in the long run since it is actually mainly microbial necromass that contributes to soil organic matter with long residence time (Miltner et al., 2012; Liang et al., 2017).

Like methane and CO_2_, the greenhouse gas nitrous oxide is also both produced and reduced by microbes and there is increasing attention on microbes that reduce nitrous oxide due to their potential for mitigating emissions. Here, the non-denitrifying nitrous oxide reducers are of special interest as they can act as sinks and reduce nitrous oxide produced by other microbes (Hallin et al., 2018). Within the nitrogen cycle, it has become increasingly evident that many microbes show greater metabolic versatility than previously thought and can have the capacity to perform several nitrogen transformation processes (Stein and Klotz, 2016). For example, the ammonia oxidizing archaea *Nitrososphaera gargensis* can gain energy from cyanate (Palatinszky et al., 2015) and the nitrite oxidizer *Nitrospira moscoviensis* has been shown to grow aerobically using hydrogen and can also oxidize organic acids while respiring nitrate (Koch et al., 2014, 2015). The latter shows that the nitrogen cycle is modular and a particular organism can be involved in different pathways. Specific pathways are also highly modular, as exemplified by the denitrification and nitrification pathways, resulting in networks of interacting organisms (Jones and Hallin, 2019). However, the idea that nitrification is always a two-step process performed by two different organisms was abandoned in 2015 when the first bacteria capable of complete ammonia oxidation (comammox) to nitrate, *Nitrospira inopinata*, was characterized (Daims et al., 2015; Van Kessel et al., 2015), although the existence of comammox had been anticipated nearly 10 years earlier (Costa et al., 2006). Similarly, microbes exploiting other nitrogen transforming reactions will be discovered. In theory, there are additional exergonic or disproportionation reactions that microbes could take advantage of and some of these would be possible as alternative reactions using known enzymes (Kuypers et al., 2018).

The discovery of missing pieces of the biogeochemical puzzle will continue, but *the overarching main grand challenge in terrestrial microbiology will remain, i.e., putting the pieces in the puzzle and connecting dynamics and composition of microbial communities with what they actually do in their natural environmental context*, as already highlighted in the Grand Challenges in 2011 (Stein and Nicol, 2011). This challenge has spurred technology development and the omics revolution has enabled a sample throughput that allows for assessing high resolution community dynamics as is required for answering ecological questions in proper spatial as well as temporal resolution (Oliverio et al., 2017; Delgado-Baquerizo et al., 2018). The functional potential and traits of uncultured microbes has been interrogated in an unprecedented way, generating information leading to the reconstruction of thousands environmentally retrieved genomes, e.g., involved in carbon cycling (Woodcroft et al., 2018). However, this “tsunami” of data is also at the basis of maybe *the currently biggest challenge in terrestrial microbiology, that being data processing, integration, and comparison*. The data amount and complexity has given rise to a huge number of bioinformatic and biostatistical tools, repositories and platforms, seriously hampering data integration and rigorous peer review of manuscripts due to lack of bioinformatic specialists. The lack of proper archiving with meta-data (e.g., soil chemistry, activity data, GHG fluxes, etc.) limits accessibility and synthesis of datasets leading to significant under exploitation of the data potential; thus, data interoperability is equally important as accessibility.

Given the amount and speed of sequencing data generated, it is also questionable whether the adoption of the “best practices in microbiome analyses” (Knight et al., 2018) can keep up. Moreover, most of this type of data represents relative abundance data, which is not quantitative, making it difficult to compare across studies and hampers inclusion of community data in models. *Acquiring quantitative data is a major challenge* in itself, not only in terrestrial microbiology but in microbial ecology in general. The ease of generating sequencing data has led to a revival of descriptive, even almost naturalist type of studies similar to what was seen in the 1990's facilitated by techniques like DGGE and TRFLP that were in fashion at that time. *It is a great challenge to ensure that research resources are wisely used to balance efforts between descriptive and hypotheses driven mechanistic research***. **We need to go from correlation to causation and deliver data that can assist in sustainable use of soils, food production and mitigation of climate change and environmental pollution. However, this requires experimentation considering multiple factors alone or in combination, executed in field or model systems allowing for extrapolation to “real world” scenarios. The latter will also require assessing multiple ecosystem functions simultaneously on microbial relevant scales. There are promising studies available working toward these goals (Wagg et al., 2014; Delgado-Baquerizo et al., 2016) and methods to study microbes in their chemical and physical soil micro-environment are under development (Baveye et al., 2018). Stable isotope labeling techniques have advanced to the level of quantitative incorporation in DNA and RNA of single taxa at high resolution (Papp et al., 2019) and by combining with high throughput sequencing allow for assessing actively interacting species in e.g., carbon and nitrogen cycling (Daebeler et al., 2014). NanoSIMS masspectrometry and Raman-based microscopy in combination with stable isotopes have even opened up for determining single cell activity at microbial relevant scales (Eichorst et al., 2015; Lee et al., 2019). However, these techniques are not yet amenable to high sample throughput. The application of machine learning techniques in terrestrial microbiology offers the data exploration capacity that will be required for dealing with the multitude of data types coming from multifactorial experiments. Functional traits of soil microbes have been connected to carbon and nitrogen cycling as well as soil physics and chemistry in this way, offering at least the mathematical tools to meet the *data integration and exploration challenge* (Hartman et al., 2017; Thompson et al., 2019).

*A never-ending grand challenge in terrestrial microbiology is the linking of microscale processes in soils to landscape scale or even to the atmosphere*. Progress in this area is urgently needed considering the need for manipulation and managing of the soil microbiome to be part of the solutions for environmental restoration, climate mitigation and sustainable agriculture. Novel modeling approaches have been developed to link microscale data to macroscale processes using mechanistic individual-based, soil-pore network models enabling for example the prediction of permafrost thaw on methane emissions (Ebrahimi and Or, 2017). Microbial explicit information, like dormancy, growth strategies, lag in responses or adaptation to environmental changes have been incorporated into global circulation models leading to better estimations of soil carbon sequestration under climate change (Wieder et al., 2015). How we can represent microbial processes into models, e.g., earth system models, need further development, and the amount of details these models can accommodate is still limited. Novel ways of computing will very likely solve some of these issues, facilitating the incorporation of complex datasets into predictive models and linking scales and also different levels of biological organization. Viruses, bacteria, archaea and eukaryotes are still predominantly studied as separate entities by different scientific communities even though we know that inter kingdom interactions are important also for biogeochemical cycling (Wagg et al., 2019) and as such, should be studied in an integrative way. *One of the greatest challenges will be to encompass the full breadth of microbial life in terrestrial systems in scientific studies and assemble the multidisciplinary teams needed to carry them out*. This is especially relevant since the elemental cycles catalyzed by various microbial groups interact, necessitating integrated studies on taxonomic as well as functional level. The nitrogen and carbon cycles are inevitably linked with many potential negative and positive interactions between nitrogen and carbon cycling microbes (Bodelier and Steenbergh, 2014), ultimately affecting agriculture and climate. Recent results demonstrating that methane consuming bacteria can be inhibited as well as stimulated by volatile chemical compounds produced by carbon cycling microbes (Veraart et al., 2018), opens up a can of worms on the level of detail that needs to be taken into account to obtain mechanistic understanding on fundamental interactions at the organisms level with consequences for global biogeochemical cycles.

Despite the many challenges in terrestrial microbiology and the large knowledge gaps that exists, *the greatest challenge of all is to translate knowledge in terrestrial microbiology into proposed solutions to environmental problems like climate change and for facilitating the transition to a sustainable economy*, and here terrestrial environments and their biota have a crucial role. Similar to the human microbiome, the importance of the soil microbiome is increasingly being acknowledged by the public, policymakers and politicians. The momentum for obtaining resources for research filling in the gaps is there. Shifting a substantial part of the efforts from omics driven gene and species inventories to fundamental microbiology, mechanistic experimentation (including field experiments), and wisely designed field studies may facilitate tackling some of the Grand Challenges.

## Author Contributions

SH and PB equally contributed to the content and writing of this manuscript.

## Conflict of Interest

The authors declare that the research was conducted in the absence of any commercial or financial relationships that could be construed as a potential conflict of interest.
